# Neurally Adjusted Ventilatory Assist Compared with Volume-Targeted and Pressure-Controlled Modes in Preterm Infants with Respiratory Distress Syndrome

**DOI:** 10.3390/jcm15062177

**Published:** 2026-03-12

**Authors:** Jiseon Park, Hannah Cho, Yeong Seok Lee, Juyoung Lee

**Affiliations:** 1 Department of Pediatrics, Korea University Anam Hospital, Seoul 02841, Republic of Korea; gm20150120@gmail.com (J.P.); hannahv2@korea.ac.kr (H.C.); 2 Department of Pediatrics, Korea University College of Medicine, Seoul 02841, Republic of Korea; 3 Department of Pediatrics, Inha University Hospital, Incheon 22332, Republic of Korea; savior_light@naver.com

**Keywords:** ventilator-induced lung injury, respiratory distress syndrome, mechanical ventilation, neurally adjusted ventilatory assist, preterm infants

## Abstract

**Background/Objectives**: Preterm infants with respiratory distress syndrome (RDS) require mechanical ventilation but risk lung injury This study compared neurally adjusted ventilatory assist (NAVA) with conventional modes regarding respiratory mechanics and clinical outcomes. **Methods**: We analyzed data from 79 preterm infants born at <32 weeks gestation who were invasively ventilated for RDS and classified into three groups: NAVA (n = 26), volume-targeted (VT; n = 29), and pressure-controlled (PC; n = 24). Respiratory parameters for 6 h post-surfactant administration and clinical outcomes were evaluated. **Results**: Baseline characteristics were similar across groups. The NAVA group demonstrated the most rapid reduction in peak inspiratory pressure over 6 h (F = 4.125, *p* = 0.023) and the fastest increase in dynamic compliance during the first 4 h (F = 3.273, *p* = 0.048). Respiratory rates were significantly lower with NAVA than with VT or PC modes, while tidal volumes were significantly higher in PC than in NAVA or VT modes. Invasive mechanical ventilation duration was shorter in NAVA (3.0 [0.9–4.9] days) than in PC modes (15.1 [0.3–38.5] days, *p* = 0.031), whereas not significantly different from that in VT modes (3.8 [0.9–13.4] days). While bronchopulmonary dysplasia or death was lower in NAVA (19.2%) than in PC modes (41.7%), the difference was not statistically significant (*p* = 0.092). **Conclusions**: NAVA resulted in the fastest reduction in ventilator-delivered pressure and earlier improvement in dynamic compliance while maintaining respiratory rates within physiological ranges and was associated with shorter ventilation duration than PC modes. However, VT modes achieved comparable respiratory parameters and ventilation durations to those achieved using NAVA.

## 1. Introduction

Respiratory distress syndrome (RDS) in preterm infants results from surfactant deficiency combined with structural and functional lung immaturity. The immediate post-surfactant period represents a physiologically dynamic transition phase characterized by rapid improvement in lung compliance, aeration redistribution, and substantial shifts in pressure–volume relationships. Inappropriate ventilatory support during this window may result in unintended overdistension and related injuries. The immature lungs of preterm infants are highly vulnerable to ventilator-induced lung injury (VILI), which encompasses barotrauma, volutrauma, atelectotrauma, biotrauma, and rheotrauma, and is further influenced by patient factors such as underlying lung disease and nutritional status [1,2,3,4].

Neurally adjusted ventilatory assist (NAVA) provides synchronized and proportional respiratory support based on the electrical activity of the diaphragm (Edi), potentially optimizing patient–ventilator interactions. By coupling ventilatory assist to Edi, NAVA may theoretically optimize RDS management by proportionally adapting support to evolving respiratory drive and limiting injurious pressure or volume delivery [5]. Previous studies have shown that NAVA can improve synchrony and oxygenation while reducing peak inspiratory pressures (PIPs), tidal volumes (TVs), and work of breathing compared with conventional modes in preterm infants [6,7,8].

However, comparative data focusing specifically on the acute post-surfactant period remain scarce, and there is currently insufficient evidence demonstrating that NAVA is superior to other contemporary modes in reducing overall VILI or improving clinical outcomes [9,10]. As volume-targeted (VT) ventilation is now widely considered a lung-protective standard over conventional pressure-controlled (PC) modes, clarification of whether NAVA offers additional physiological or clinical advantages beyond VT strategies is essential. Therefore, in this observational study, we compared the respiratory mechanics and clinical outcomes among NAVA, VT, and PC modes in preterm infants with RDS during the acute lung recovery period following surfactant administration.

## 2. Materials and Methods

### 2.1. Study Design and Participants

This was an observational cohort study using prospectively collected data; no randomization was performed and all ventilatory management decisions were made at the discretion of the treating clinicians. The study was conducted in two level III neonatal intensive care units: Inha University Hospital (Incheon) and Korea University Anam Hospital (Seoul), Republic of Korea.

**Figure 1:**
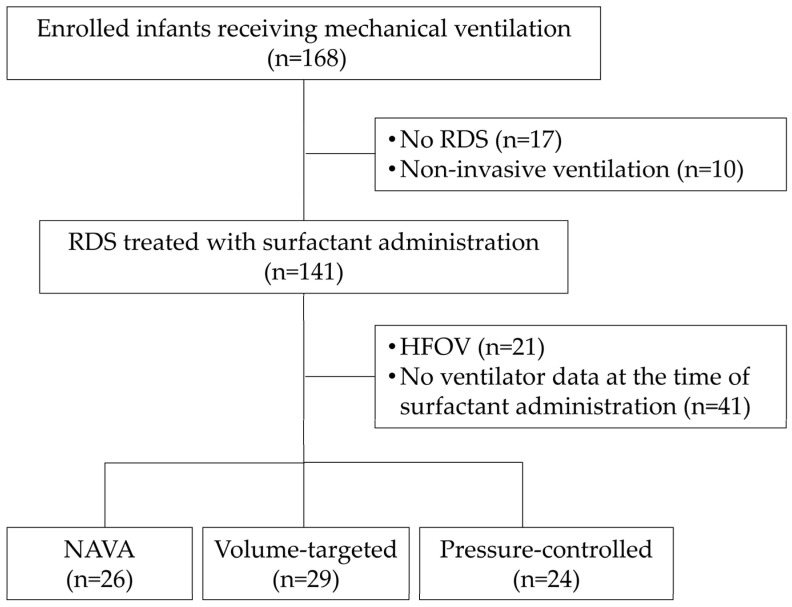
Study participants. RDS, respiratory distress syndrome; HFOV, high-frequency oscillatory ventilation; NAVA, neurally adjusted ventilatory assist.

Among 168 preterm infants born between February 2021 and June 2024, infants were eligible if they: (1) were born at less than 32 weeks’ gestation; (2) had clinically confirmed RDS; (3) received invasive mechanical ventilation using Servo-n ventilators (Getinge, Solna, Sweden); and (4) received surfactant therapy. A total of 79 infants met all criteria and were included in the final analysis. Infants were excluded if they: did not have RDS (n = 17), received non-invasive ventilation only (n = 10), received high-frequency oscillatory ventilation (n = 21), or did not have ventilator data at the time of surfactant administration (n = 41) (Figure 1). Although formal radiographic severity scoring was not applied, all enrolled infants required intubation and surfactant therapy, indicating moderate-to-severe disease requiring invasive support. The ventilation mode was selected by the attending physician based on clinical assessment. VT modes were typically used as first-line invasive ventilation; PC modes were chosen in cases of high endotracheal tube leakage, and NAVA was sometimes selected initially in infants with adequate spontaneous respiratory effort. Surfactant (poractant alfa, 200 mg/kg initial dose) was administered via an endotracheal tube according to the institutional protocol. Repeat dosing (100 mg/kg) was considered if persistent fraction of inspired oxygen (FiO_2_) > 0.30–0.40 was required and there was radiographic evidence of ongoing RDS 6–12 h after the first surfactant therapy.

### 2.2. Data Collection and Ventilator Settings

Respiratory parameters were collected for 6 h following surfactant administration to capture the period of acute changes in lung compliance, pressure, and volume after treatment. All infants received routine caffeine citrates. Sedation is not routinely used for this age group (preterm infants).

VT modes: VT modes (volume control and pressure-regulated volume control) used a target TV of 4–6 mL/kg, with a typical initial setting of 5 mL/kg for most infants (Table S1). TV was subsequently adjusted based on serial arterial or capillary blood gases (target pH of 7.25–7.35, PaCO_2_ of 5–7 kPa) and clinical assessment of chest expansion.

PC modes: PC modes included synchronized intermittent mandatory ventilation (SIMV), pressure support ventilation, and SIMV with pressure support. Initial PIP was set at 16–20 cmH_2_O, with positive end expiratory pressure (PEEP) initiated at approximately 5 cmH_2_O and adjusted individually based on blood gas results, oxygen requirement and clinical response. Backup rates for SIMV were generally 30–50 breaths/min with inspiratory times of 0.3–0.4 s (Table S1). PIP was titrated according to blood gases and chest wall expansion.

NAVA mode: For NAVA, the Edi trigger level was set at 0.5 μV above the minimum Edi, and the NAVA level was set between 1.0 and 2.5 cmH_2_O/μV to target peak Edi values of 5–15 μV (Table S1). PEEP, backup ventilation in pressure control mode, and FiO_2_ were adjusted based on the individual response with a target peripheral oxygen saturation of 90–95%. The maximum PIP safety limit was set at 30–40 cmH_2_O.

Weaning criteria: Infants were considered for weaning when they demonstrated adequate spontaneous respiratory effort, improving lung compliance, and acceptable blood gas values (pH ≥ 7.25, PaCO_2_ ≤ 7 kPa).

### 2.3. Measurements and Outcomes

PIP and expiratory TV were measured using a Y-piece hot-wire flow sensor at the proximal end of the patient circuit. Dynamic compliance data were automatically calculated and recorded by the Servo-n ventilator (Getinge, Solna, Sweden). The device utilizes an automated algorithm to compensate for circuit compliance. By subtracting the volume lost to circuit expansion, the ventilator provides a more precise measurement of the patient’s actual TV and lung compliance. This technical compensation results in the reported dynamic compliance possibly appearing higher than raw (TV/ΔP [PIP-PEEP]) estimations.

All respiratory parameters were recorded automatically every minute and exported via USB using Servo Record Viewer version 1.0 (Maquet Critical Care AB, Getinge, Gothenburg, Sweden).

The primary outcome was the change in PIP over the 6 h period after surfactant administration. The secondary outcomes were changes in expiratory TV, respiratory rate, and dynamic lung compliance measured by the ventilator. Clinical data included gestational age, birth weight, sex, Apgar scores, small for gestational age status, oligohydramnios, premature rupture of membranes (>18 h), histological chorioamnionitis, antenatal steroids (any and complete course), airway problems (documented vocal cord injury, laryngeal stenosis, or tracheal abnormalities), meconium aspiration syndrome, patent ductus arteriosus requiring medical or surgical treatment, timing of surfactant administration and its doses, durations of invasive and non-invasive mechanical ventilation, hospital stay, air leaks, pulmonary hemorrhage, bronchopulmonary dysplasia (BPD; oxygen need at 36 weeks postmenstrual age), intraventricular hemorrhage (IVH; grade ≥ 3), retinopathy of prematurity requiring treatment, and death.

### 2.4. Ethical Approval

The study was approved by the Institutional Review Boards of Inha University Hospital, Incheon, Republic of Korea (2021-04-034, approved on 3 April 2021), and Korea University Anam Hospital, Seoul, Republic of Korea (K2024AN0176, approved on 11 April 2024), and conducted in accordance with the Declaration of Helsinki. Written informed consent was obtained from the parents before collecting ventilator data.

### 2.5. Statistical Analysis

Categorical variables are presented as numbers (percentages), and continuous variables as medians (interquartile ranges) or means ± standard deviations. Baseline characteristics among the three groups were compared using Mann–Whitney U or Kruskal–Wallis tests with Bonferroni correction for continuous variables and Fisher’s exact test for categorical variables.

Respiratory parameters over the 6 h period were analyzed using repeated-measures analysis of variance (RM-ANOVA), with time as a within-subject factor and ventilation mode as a between-subject factor, to assess both overall time-course changes and differences in trajectories among groups. Time to successful extubation was analyzed using the Kaplan–Meier method, and differences between the groups were compared using the log-rank test. Statistical analyses were performed using SPSS v.27.0 (IBM Corp., Armonk, NY, USA). A *p*-value < 0.05 was considered statistically significant.

## 3. Results

### 3.1. Study Population and Baseline Characteristics

Of the 168 preterm infants initially screened, 79 fulfilled the inclusion criteria and were assigned to NAVA (n = 26), VT (n = 29), or PC (n = 24) groups based on the ventilatory mode used (Figure 1). The ventilator mode remained unchanged during the 6 h observation period.

Baseline demographic and clinical characteristics did not differ significantly among groups (Table 1). Median gestational age was approximately 28–29 weeks, and median birth weight ranged from 1085 to 1300 g. The sex distribution, Apgar scores, antenatal steroid exposure, and perinatal factors, including chorioamnionitis and premature rupture of membranes, were comparable across the groups. Surfactant was administered at a median of 3.0–3.6 min after birth in all groups, that is, before the 5 min Apgar assessment. There was no difference in the number of patients who received repeated of surfactant therapy, with one in each of the three groups.

### 3.2. Respiratory Parameters During the 6 h Post-Surfactant Period

**Figure 2:**
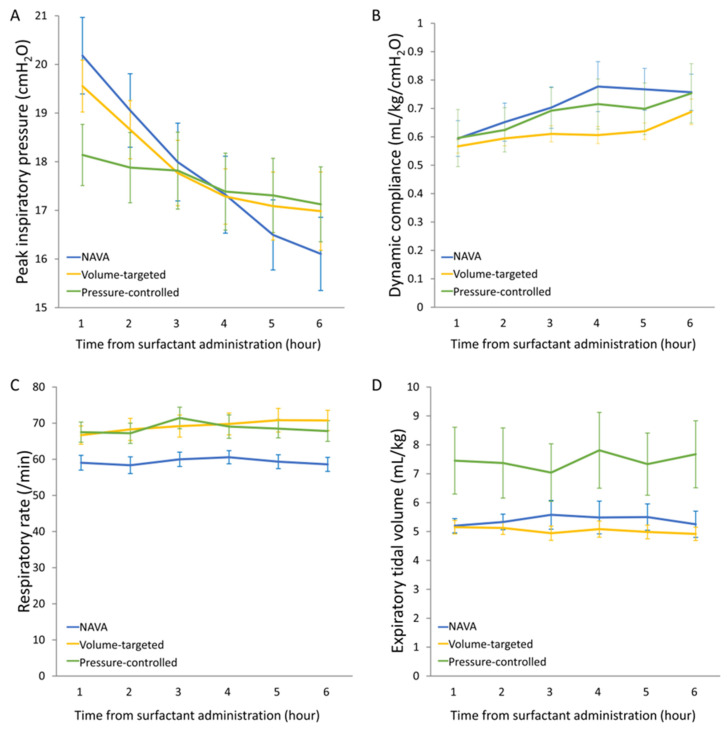
Respiratory parameters following surfactant administration. (**A**), peak inspiratory pressure; (**B**), dynamic compliance; (**C**), respiratory rate; (**D**), expiratory tidal volume. Values are the mean ± SEM. Repeated-measures analysis of variance was used. NAVA, neurally adjusted ventilatory assist.

Peak inspiratory pressure: NAVA showed the most rapid reduction in PIP over the 6 h following surfactant administration (time-by-group interaction: F = 4.125, *p* = 0.023; Figure 2A). Initial mean PIP values at 1 h were similar (NAVA: 20.1 cmH_2_O; VT: 19.4 cmH_2_O; PC: 18.1 cmH_2_O), however PIP declined most steeply in the NAVA group, reaching 15.8 cmH_2_O at 6 h compared with 16.9 cmH_2_O in both the VT and PC groups.

Dynamic compliance: Dynamic compliance increased in all groups following surfactant administration, but the improvement was more pronounced in NAVA during the first 4 h (F = 3.273, *p* = 0.048 for time-by-group interaction between 1 and 4 h; Figure 2B) compared with the VT and PC groups. NAVA exhibited the steepest early gain in compliance, consistent with more effective lung recruitment and stabilization.

Respiratory rate: Respiratory rates were significantly lower in NAVA (58.6 ± 1.9 breaths/min) compared with VT (70.8 ± 2.8 breaths/min, *p* = 0.028) and PC (67.8 ± 2.9 breaths/min, *p* = 0.041) (Figure 2C). The rates remained relatively stable throughout the 6 h in the NAVA group, indicating that the PIP reduction with NAVA was not achieved at the expense of compensatory tachypnea.

Tidal volume: Expiratory TV differed significantly across modes (Figure 2D). PC modes generated higher TVs (7.7 ± 1.2 mL/kg) compared to NAVA (5.3 ± 0.5 mL/kg, *p* = 0.017) and VT (5.0 ± 0.2 mL/kg, *p* = 0.012). VT modes maintained TVs at around 5 mL/kg for 6 h, consistent with the targeted range, while NAVA also maintained relatively stable TVs in a similar range. PC modes, in which TV is pressure-dependent, produced larger TVs as the lung compliance improved, reflecting the underlying pressure–volume relationship.

### 3.3. Clinical Outcomes

**Figure 3:**
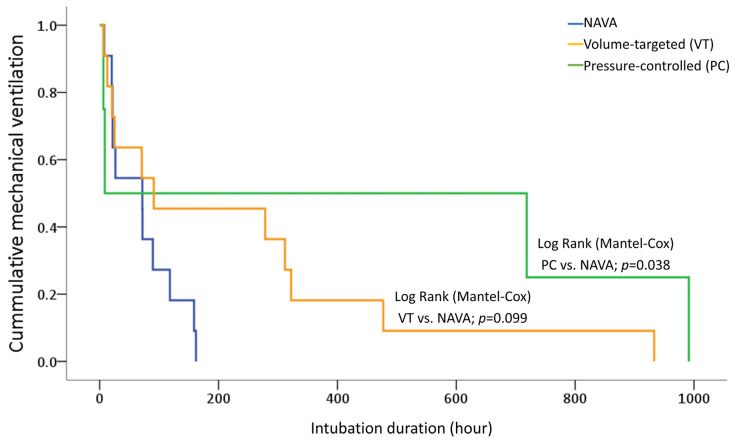
Kaplan–Meier curves for invasive mechanical ventilation duration according to the ventilatory mode. Statistical significance was determined using the log-rank (Mantel–Cox) test. NAVA, neurally adjusted ventilatory assist.

Invasive mechanical ventilation duration was significantly shorter in NAVA (median 3.0 days, IQR: 0.9–4.9) compared with PC modes (median: 15.1 days, IQR: 0.3–38.5, *p* = 0.031). The VT group had a median duration of 3.8 days (IQR: 0.9–13.4), which was not significantly different from NAVA (*p* = 0.365) (Table 2). In the Kaplan–Meier analysis, the NAVA group demonstrated a significantly shorter intubation duration compared with the PC group (log-rank test, *p* = 0.038). Although the NAVA group also showed a trend toward earlier extubation compared with the VT group, the difference was not significant (log-rank test, *p* = 0.099). These results suggest that NAVA may facilitate a faster transition to non-invasive respiratory support in critically ill neonates with RDS (Figure 3).

Non-invasive ventilation duration, length of hospital stay, and rates of pneumothorax, pulmonary hemorrhage, severe IVH (grade ≥ 3) or death, and treated retinopathy of prematurity did not differ significantly among groups. BPD at 36 weeks postmenstrual age or death was observed in 19.2% (NAVA), 27.6% (VT), and 41.7% (PC) of the groups. Although the occurrences of BPD or death were numerically lower with NAVA than with PC modes, this difference did not reach statistical significance after Bonferroni correction (*p* = 0.092).

## 4. Discussion

This observational cohort study is, to our knowledge, the first to directly compare NAVA with both VT and PC modes in preterm infants with RDS during the acute post-surfactant period, focusing on temporal changes in ventilatory parameters and short-term clinical outcomes.

NAVA was associated with faster PIP decline and earlier improvement in dynamic compliance than VT or PC modes while maintaining lower respiratory rates and TVs comparable to VT modes. Fixed pressure strategies in PC modes may overshoot delivered TVs, whereas VT modes constrain volume but do not directly synchronize neural timing. NAVA integrates both proportionality and synchrony, which may explain the observed steeper PIP decline and early compliance improvement. PC modes delivered substantially higher TVs, which may contribute to the longer invasive ventilation duration observed in this group given the known association between larger TV and volutrauma in preterm lungs [11,12].

VT modes achieved invasive ventilation durations similar to NAVA and did not differ significantly in other major clinical outcomes, suggesting that contemporary volume-targeted strategies can provide clinical results comparable to NAVA when carefully implemented. This attenuates the strength of any conclusion regarding NAVA’s clinical superiority and indicates that its primary benefits may be physiological rather than clearly outcome-changing in this context.

VILI represents a complex interplay of mechanical stress (barotrauma, volutrauma, and atelectotrauma), flow-related injury (rheotrauma), and inflammatory/biochemical responses, modulated by patient-specific factors such as lung immaturity and comorbid lung disease [1,11,12,13,14,15,16,17,18,19]. Although improving synchrony and proportional unloading with NAVA theoretically targets several of these mechanisms, this study did not directly measure synchrony indices, inflammatory markers, or long-term pulmonary outcomes, and therefore cannot confirm that NAVA reduces overall VILI burden [19,20,21].

Our results are broadly consistent with prior randomized trials and systematic reviews reporting the physiological advantages of NAVA (lower PIP and TV, and reduced work of breathing), though these had limited or inconsistent evidence of superior long-term outcomes compared with other modern ventilatory modes [6,7,8,9,22,23,24]. The comparable performance of VT modes in our cohort aligns with this pattern and supports the notion that well-implemented volume-targeted ventilation remains an effective lung-protective strategy in preterm infants with RDS [11,12,20,21,25,26].

This study has some important limitations. The non-randomized design and clinician-selected ventilation modes introduce possible selection bias, particularly as NAVA was sometimes chosen for infants with better spontaneous respiratory drive. RDS severity at enrollment was not formally graded, and only the data from a 6 h observation period was analyzed, limiting insights into longer-term trajectories. In addition, specific mode subtypes within VT and PC categories may also restrict generalizability, and multiple comparisons raise the possibility of type I error despite Bonferroni correction.

## 5. Conclusions

NAVA, compared with PC modes, resulted in faster PIP reduction and earlier improvement in dynamic compliance during acute RDS recovery in preterm infants while maintaining lower respiratory rates and avoiding excessive TVs. Invasive mechanical ventilation duration was shorter with NAVA than with PC modes; however, VT ventilation achieved durations comparable to NAVA, with similar short-term outcomes. These findings suggest that NAVA is a physiologically attractive option for ventilatory support in preterm infants with RDS but do not provide definitive evidence that NAVA confers superior clinical benefit or reduces overall VILI when compared with well-implemented volume-targeted strategies. Well-designed prospective randomized trials with detailed assessments of VILI mechanisms and long-term respiratory and neurodevelopmental outcomes are needed to clarify the role of NAVA and identify infants most likely to benefit.

## Figures and Tables

**Table 1:** Baseline characteristics.

	NAVA (n = 26)	Volume-Targeted (n = 29)	Pressure-Controlled (n = 24)	*p*-Value
Gestational age, wk	28^+6^ (26^+5^–30^+5^)	28^+6^ (25^+0^–31^+5^)	29^+5^ (27^+4^–31^+1^)	0.801
Birth weight, g	1085 (820–1670)	1300 (815–1705)	1215 (900–2180)	0.906
Sex, male:female	10:16 (38.5:61.5)	14:15 (48.3:51.7)	8:16 (33.3:66.7)	0.267
1-min Apgar score	2 (1–3)	3 (2–5)	2 (1–5)	0.731
5-min Apgar score	5 (4–6)	6 (4–7)	4 (3–6)	0.300
Vaginal delivery	4 (15.4)	3 (10.3)	2 (8.3)	0.866
Small for gestational age	3 (11.5)	2 (6.9)	2 (8.3)	0.911
Oligohydramnios	4 (15.4)	3 (10.3)	1 (4.2)	0.373
PROM > 18 h	4 (15.4)	6 (20.7)	3 (12.5)	0.752
Chorioamnionitis	2 (7.7)	7 (24.1)	3 (12.5)	0.167
Antenatal steroids, any	20 (76.9)	19 (65.5)	21 (87.5)	0.754
Antenatal steroids, complete	17 (65.4)	17 (58.6)	15 (62.5)	0.938
Meconium aspiration syndrome	1 (3.8)	1 (3.4)	0	0.609
Patent ductus arteriosus, treated	10 (38.5)	11 (37.9)	7 (29.2)	0.821
Surfactant therapy, min after birth	3.0 (1.2–28.8)	3.6 (1.2–81)	3.0 (1.2–120.6)	0.896
Repeat of surfactant therapy	1 (3.8)	1 (3.4)	1 (4.2)	0.994

Values presented are numbers (%) or medians (interquartile range). Fisher’s exact test or Kruskal–Wallis test with Bonferroni correction was used. NAVA, neurally adjusted ventilatory assist; PROM, premature rupture of membrane.

**Table 2:** Clinical outcomes.

	NAVA (n = 26)	Volume-Targeted (n = 29)	Pressure-Controlled (n = 24)	*p*-Value ^1^	*p*-Value ^2^
Invasive MV duration, d	3.0 (0.9–4.9)	3.8 (0.9–13.4)	15.1 (0.3–38.5)	0.365	0.031
Non-invasive MV duration, d	20.0 (4.8–45.0)	27.8 (13.0–58.9)	41.8 (12.9–60.2)	0.571	0.056
Hospital stay, d	58 (37–97)	52 (31–76)	87 (58–106)	0.519	0.343
Pneumothorax	1 (3.8)	1 (3.4)	0	0.609	>0.999
Pulmonary hemorrhage	1 (3.8)	2 (6.9)	0	0.399	>0.999
BPD or death	5 (19.2)	8 (27.6)	10 (41.7)	0.543	0.092
IVH (grade ≥ 3) or death	2 (7.7)	4 (13.8)	0	0.424	0.733
ROP (treated)	3 (11.5)	4 (13.8)	6 (25.0)	0.661	0.243
Death	1 (3.8)	1 (6.9)	0	>0.999	>0.999

Values are numbers (%) or medians (interquartile range). Fisher’s exact tests or Mann–Whitney U tests were used. ^1^ NAVA group vs. volume-targeted group; ^2^ NAVA group vs. pressure-controlled group. NAVA, neurally adjusted ventilatory assist; MV, mechanical ventilation; BPD, bronchopulmonary dysplasia at 36 weeks postmenstrual age; IVH, intraventricular hemorrhage; ROP, retinopathy of prematurity.

## Data Availability

The data supporting the findings of this study are not publicly available due to participant confidentiality but are available from the corresponding author upon reasonable request.

## Supplementary Materials

The following supporting information can be downloaded at: https://www.mdpi.com/article/10.3390/jcm15062177/s1, Table S1: Initial ventilator settings.

## Abbreviations

The following abbreviations are used in this manuscript:

RDS	respiratory distress syndrome
VILI	ventilator-induced lung injury
NAVA	neurally adjusted ventilatory assist
Edi	electrical activity of the diaphragm
VT	volume-targeted
PC	pressure-controlled
PIP	peak inspiratory pressure
TV	tidal volume
FiO_2_	fraction of inspired oxygen
BPD	bronchopulmonary dysplasia
IVH	intraventricular hemorrhage
SIMV	synchronized intermittent mandatory ventilation
PEEP	positive end expiratory pressure
