# Supplementary material for: Neurally Adjusted Ventilatory Assist Compared with Volume-Targeted and Pressure-Controlled Modes in Preterm Infants with Respiratory Distress Syndrome
Source: J Clin Med. 2026 Mar 12;15(6):2177. doi: 10.3390/jcm15062177 (PMC13026100; doi:10.3390/jcm15062177)
Supplement: Supplementary file 1 [file jcm-15-02177-s001.zip › jcm-4174430-supplementary.pdf]

Supplementary Materials

Table S1. Initial ventilator settings

	**NAVA (n=26)**	**Volume-targeted (n=29)**	**Pressure-controlled (n=24)**	***p*-Value**
Initial FiO_2 before surfactant therapy	0.5 (0.45–0.55)	0.5 (0.4–0.55)	0.5 (0.4–0.6)	0.975
PEEP, cmH_2O	5 (5–5.5)	5 (5–6)	5 (5–5.5)	0.506
Backup frequency, /min	45 (40–50)	45(45–50)	45 (42.5–47.5)	0.895
Backup inspiratory time, sec	0.32 (0.30–0.34)	0.34 (0.32–0.35)	0.32 (0.30–0.34)	0.636
Peak inspiratory pressure, cmH_2O	NA	NA	18.2 (17.5–18.9)	NA
Tidal volume, mL/kg	NA	5.0 (5.0–5.28)	NA	NA
NAVA level	1.7 (1.5–1.8)	NA	NA	NA

Values are presented as median (interquartile range).

Kruskal–Wallis test with Bonferroni correction were used.

NAVA, neurally adjusted ventilatory assist; FiO_2, fraction of inspired oxygen; PEEP, positive end-expiratory pressure; NA, non-applicable.
